# Comparison of Metabolites and Main Nutritional Components between Uncooked and Cooked Purple Rice

**DOI:** 10.3390/metabo13091018

**Published:** 2023-09-15

**Authors:** Wenfei Gu, Yuehong Peng, Ruizhi Wang, Runnan Wang, Han Wu, Jinyan Zhu, Xinhua Ni, Qiangqiang Xiong

**Affiliations:** 1 Jiangsu Key Laboratory of Crop Genetics and Physiology/Jiangsu Key Laboratory of Crop Cultivation and Physiology, Agricultural College of Yangzhou University, Yangzhou 225009, China; 2 Jiangsu Co-Innovation Center for Modern Production Technology of Grain Crops, Yangzhou University, Yangzhou 225009, China; 3 Jiangsu Zijiang Ecological Agriculture Co., Ltd., Yangzhou 212200, China

**Keywords:** purple rice, cooking, metabolomics, amino acids, flavonoids, antioxidant properties

## Abstract

Cooking can lead to varying degrees of nutrient loss in purple rice. For this investigation, two varieties of purple rice (YZN1 and YZ6) were chosen as the focal points to explore the metabolites associated with rice nutrition post cooking using nontargeted and targeted metabolomics techniques. The results showed that after cooking the two purple rice varieties, the contents of the flavonoids; OPC; TP; total antioxidant capacity; and K, Na, Fe, Mn, Zn, Cu, Ca, and Mg significantly decreased. Compared with YZN1U (YZN1 uncooked), the amino acid and mineral element contents in YZN1C (YZN1 cooked) decreased to varying degrees. After cooking YZ6, the contents of seven amino acids significantly decreased. Following the preparation of purple rice, the metabolites primarily engaged in the pathways of flavonoid synthesis and flavone and flavonol synthesis. Flavonoids, total antioxidant capacity, mineral elements, and amino acids showed a strong correlation with delphinidin and luteolin. The ROC analysis demonstrated that the value of the area under the curve for delphinidin and luteolin was 1 when comparing YZ6C (YZ6 cooked) and YZ6U (YZ6 uncooked), as well as YZN1C and YZN1U. Delphinidin and luteolin can be used as potential biomarkers of nutrient loss after cooking purple rice. This study holds significant implications for the balanced nutrition and healthy development of human dietary grains.

## 1. Introduction

Colored rice refers to rice varieties that have a distinct coloration, and it is a precious and distinctive rice resource in China [1]. The presence of abundant anthocyanins accumulated in the husk and bran of rice grains gives them colors such as purple, dark purple, and purplish red, resulting in purple rice varieties. Colored rice varieties not only come in a variety of colors but also offer a rich source of nutrients, including proteins, fats, starch, polyphenols, flavonoids, anthocyanins, dietary fiber, vitamins, and essential minerals such as Fe, Zn, Ca, and P. These nutrients have been proven to be beneficial for human health [2,3]. The consumption of colored rice can help supplement essential nutrients in the human body, thereby enhancing immune function and preventing diseases [4,5]. In a study, healthy volunteers who consumed yogurt containing 0.25% purple rice anthocyanins demonstrated significant inhibition of postprandial glucose levels and improved plasma antioxidant capacity [6]. The ancient Chinese medical book compendium of Materia Medica recorded that purple rice has various medicinal properties, including tonifying the spleen and warming the liver, nourishing yin and replenishing the kidneys, promoting blood circulation, and improving vision. It is also believed to have benefits such as warming the stomach, treating deficiency-cold diarrhea, relieving thirst, reducing frequent urination, and preventing spontaneous sweating [7]. In recent years, as the overall standard of living has improved, individuals have grown more interested in the nutritional content and health advantages of the food they consume. Consequently, purple rice, which possesses enhanced nutritional and medicinal properties along with health advantages, has become increasingly popular among both domestic and international consumers. During the cooking process, the nutritional elements and antioxidant characteristics of uncooked purple rice, including polyphenols, proteins, starch, flavonoids, Fe, and Ca, experience alterations caused by factors such as soaking, elevated temperature, and increased pressure [8].

Metabolomics analysis is an important strategy for the analysis of nutrients at the molecular level. The commercial maturity of rambutan usually depends on the color and edible quality of the peel. Researchers compared and analyzed the three maturation stages of the two varieties of rambutan and identified 3,4-diisopropyl shikimic acid as a potential biomarker of taste [9]. Identification of metabolic markers allows rambutan to be harvested at a time when quality and appearance achieve an optimal balance [10]. Compared with cow’s milk, the production level of goat’s milk is lower, and the price is higher. Therefore, cow milk with similar physical and chemical properties may be mixed into goat milk. Metabolomics methods can effectively distinguish milk from different species and can also be used to detect milk adulteration. Previous studies have identified bovine-specific proteins, namely, SCGB1D, β-lactoglobulin, and GlyCam1, as well as the metabolites uric acid and n-formylkynuretic acid, that can be used to detect milk adulteration as low as 1% in goat milk [11]. Therefore, metabolic markers not only play an important role in the identification of food quality but also have great application value in food adulteration traceability [12].

Metabolomics is a relatively recent discipline that emerged following genomics and proteomics. The analysis of metabolite levels in biological systems is the main focus of studying how organisms respond to external stimuli in a multidimensional and dynamic manner. Metabolomics has found wide applications in various fields, including agriculture and food science [13,14]. Over the past few years, there has been a gradual utilization of metabolomics in examining the nutritional value of agricultural products [15]. Research has been conducted on the impact of milling and processing on the nutritional metabolites of purple rice [2]. Furthermore, studies have been conducted on the detection of metabolite biomarkers associated with antioxidant characteristics during various growth phases of purple rice grains [16]. Currently, there is a shortage of research comparing the nutritional makeup and variations in metabolites of purple rice prior to and following the cooking process. Studying the variations in nutritional content and metabolites following the preparation of purple rice could offer valuable knowledge regarding the nutritional significance of colored rice post cooking.

This study aims to examine the alterations in nutritional components and associated metabolites of purple rice before and after the cooking process. The investigation specifically concentrates on two purple rice varieties (YZN1 and YZ6) developed by Yangzhou University. The analysis will include the determination of total phenolics (TPs), total flavonoids (TFs), oligomeric proanthocyanidins (OPCs), total sugars (carbohydrates), total starch, proteins, fats, ATP, dietary fiber, ash content, and mineral element content in the rice grains before and after cooking. By investigating the changes in nutritional substances and metabolites after cooking with purple rice, this study aims to provide new insights into the cultivation and utilization of high-nutrient colored rice. Further knowledge about changes in nutritional elements and compounds following the cooking process can aid in the creation of novel strategies to enhance the nutritional advantages of colored rice.

## 2. Materials and Methods

### 2.1. Plant Materials and Growth Conditions

YZN and YZ6, two purple rice varieties developed by Yangzhou University, were utilized as the experimental materials in this research. Pictures of the two types of purple rice are shown in Figure S1. Different types of rice were grown at the Shatou Base, which is situated in Yangzhou City, Jiangsu Province, China, and belongs to Yangzhou University. Each hole was filled with four rice seedlings, with a gap of 30 × 12 cm between the rows and plants. Three replications were used for planting each type of rice variety.

### 2.2. Sample Collection

At the maturity stage of rice, a total of 300 g of unpolished rice grains was collected from each of the two purple rice cultivars. The rice grains were then rapidly frozen using liquid nitrogen. Two portions were created for each rice variety, with three biological replicates taken for each. Rice is cooked in a ratio of 1:1.5 rice to water. The steaming time of purple rice is 30 min, with 10 min of insulation, and the cooking temperature of purple rice is 100 °C. The uncooked rice grains of the two varieties served as the control groups and were labeled YZN1U (YZN1 uncooked) and YZ6U (YZ6 uncooked). The cooked rice grains of the two varieties were labeled YZN1C (YZN1 cooked) and YZ6C (YZ6 cooked).

### 2.3. Evaluation of Physiological and Biochemical Parameters

The measurement of flavonoids [17] in rice grain samples was conducted using assay kits from Suzhou Michy Biomedical Technology Co., Ltd. (Suzhou Michy Biomedical Technology Co., Ltd., Suzhou, China). The contents of OPC [17], TP [18], and total antioxidant capacity (measured using the ABTS method, DPPH method, and FRAP method) [18,19] were also determined. The methods employed for determination also included the measurement of total starch (TSA), total sugar (TSU), and cellulose. The detailed procedures are described below.

Procedure for measuring flavonoid content: The sample was dehydrated until it reached a consistent weight, ground into a fine powder and filtered using a 40-mesh sieve. The sample was weighed, and approximately 0.05 g was combined with 1 mL of ethanol solution containing 60% ethanol. Next, the blend was subjected to extraction by shaking at a temperature of 60 °C for a duration of 2 h. Following centrifugation at 10,000× *g* and a temperature of 25 °C for 10 min, the resulting liquid above the sediment was gathered and utilized for subsequent analysis.

The process for determining the total starch (TSA) involved weighing approximately 0.1 g of a fresh sample and grinding it finely in a mortar. Next, 1 mL of reagent one was introduced into the mortar and meticulously blended until a slurry was formed. The mixture was moved to an EP tube and underwent extraction in a water bath at a temperature of 80 °C for 30 min. Following centrifugation at a speed of 3000× *g* and a temperature of 25 °C for 5 min, the liquid above was discarded, while the solid residue was kept. To the precipitate, 1 mL of distilled water and 0.3 mL of reagent two were added, followed by vigorous shaking to ensure thorough mixing. Next, the combination was exposed to a water bath at a temperature of 95 °C for a duration of 20 min while shaking intermittently (3–5 occasions). Following the cooling process, the supernatant was obtained through centrifugation to facilitate subsequent analysis.

Procedure for measuring cellulose content: Approximately 0.05 g (or 0.1 g for fresh samples) of the sample was weighed and combined with 1 mL of 80% ethanol to create a uniform mixture. The slurry was transferred to an EP tube and subjected to a 30 min extraction in an 80 °C water bath. Once the tube reached room temperature, it was subjected to centrifugation at a force of 10,000× *g* and a temperature of 25 °C for a duration of 2 min. Subsequently, the liquid above the sediment was removed. To the precipitate, 1 mL of pure acetone was added, followed by vortexing for 2 min. Afterward, the tube underwent centrifugation at a speed of 10,000× *g* and a temperature of 25 °C for a duration of 2 min, after which the liquid above was removed. To the remaining precipitate, 1 mL of reagent one (used to remove starch) was added. Following vortexing for 2 min, the tube was immersed for a duration of 15 h. Subsequently, the supernatant was discarded after centrifuging at 10,000× *g* and 25 °C for 2 min. One aliquot of α-amylase solution was introduced into the precipitate and incubated in a water bath at 65 °C for a duration of 30 min. The tube was then centrifuged to remove the supernatant. One of the glucoamylase solutions was added to the remaining precipitate and incubated in a water bath at 60 °C for 16 h. After centrifugation to eliminate the supernatant, the aforementioned procedures were repeated. Reagent three was added to the precipitate, thoroughly mixed, and left to stand for 30 min. Next, the tube was subjected to centrifugation at a speed of 10,000× *g* and a temperature of 25 °C for a duration of 10 min. Then, 50 μL of the supernatant was collected and combined with 200 μL of distilled water (the dilution factor can be modified according to initial experimental findings). Afterward, the blend was subjected to centrifugation at a force of 10,000 times the acceleration due to gravity and a temperature of 25 °C for a duration of 10 min. Subsequently, the resultant liquid above the sediment was utilized for subsequent examination.

Method for TSU determination: The sample was dried and powdered. A homogenized slurry was created by combining approximately 0.02 g of the sample with 2 mL of the extraction solvent after weighing. The slurry was transferred to a centrifuge tube and placed in a tightly sealed 90 °C oven for 3 h for hydrolysis. Following the cooling process, the tube underwent centrifugation at a speed of 10,000× *g* for a duration of 10 min. For measurement, the supernatant was diluted by a factor of 10 using a mixture of 100 μL of supernatant and 900 μL of distilled water.

### 2.4. Determination of Plant Element Content

The analysis of plant components such as P, K, Na, Fe, Mn, Zn, Cu, Ca, and Mg was performed using inductively coupled plasma-mass spectrometry/atomic emission spectrometry (ICP-MS/AES) (Thermo Fisher Scientific, Waltham, MA, USA).

### 2.5. Metabolite Extraction and Ultrahigh-Performance Liquid Chromatography-Mass Spectrometry (UHPLC-MS/MS)

The methods for extracting and determining metabolites refer to Xiong et al. (2022) [5]. The metabolites were separated using chromatography on a Thermo UHPLC system that had an ACQUITY UPLC HSS T3 (100 × 2.1 mm i.d., 1.8 µm; Waters, Milford, CT, USA) attached. Data were gathered using a Thermo UHPLC-Q Exactive HF-X mass spectrometer that was fitted with an electrospray ionization (ESI) source. The instrument was operated in either positive or negative ion mode. The ideal parameters were established as follows: heater temperature set at 425 °C; capillary temperature at 325 °C; sheath gas flow rate of 50 arb; Aux gas flow rate of 13 arb; ion-spray voltage floating (ISVF) at −3500 V in negative mode and 3500 V in positive mode; and normalized collision energy ranging from 20 to 40 to 60 V for MS/MS. The complete MS resolution was 60,000, while the resolution for MS/MS was 7500. Data were acquired using the data-dependent acquisition (DDA) mode. The identification was conducted across a range of masses from 70 to 1050 *m*/*z*.

### 2.6. Metabolite Statistical Analysis

Following UPLC-MS analyses, the unprocessed data were brought into Progenesis QI 2.3 (Nonlinear Dynamics, Waters, Milford, MA, USA) for identifying and aligning peaks. The preprocessing outcomes produced a data matrix containing the retention time (RT), the mass-to-charge ratio (*m*/*z*) measurements, and the intensity of the peaks. Retained metabolites were those that were detected in at least 80% of any set of samples. The metabolites were identified through the analysis of their mass spectra, accurate mass, MS/MS fragment spectra, and isotope ratio difference. This identification was achieved by searching trustworthy biochemical databases such as the Human Metabolome Database (HMDB) available at http://www.hmdb.ca/ (accessed on 7 March 2023) and the Metlin database accessible at https://metlin.scripps.edu/ (accessed on 7 March 2023).

The Majorbio Cloud Platform (https://cloud.majorbio.com, accessed on 7 March 2023) [20] was used to perform a multivariate statistical analysis with the ropls (Version 1.6.2, http://bioconductor.org/packages/release/bioc/html/ropls.html, accessed on 7 March 2023) R package from Bioconductor. Before performing PCA, all the metabolite variables were normalized to have unit variances. Before performing OPLS-DA, Pareto scaling was applied to scale all the metabolite variables. To prevent overfitting, the interpretability and predictability of the model were assessed using the model parameters R2 and Q2. The calculation of variable importance in the projection (VIP) was performed within the OPLS-DA model. Paired Student’s *t* test was used to estimate *p* values in a single-dimensional statistical analysis. Differential metabolites (DMs) were determined by considering VIP values above 1 and *p* values below 0.05. The ropls R package (Version 1.6.2, http://bioconductor.org/packages/release/bioc/html/ropls.html, accessed on 7 March 2023) from Bioconductor was utilized to conduct multivariate statistical analysis on the Majorbio Cloud Platform (https://cloud.majorbio.com, accessed on 7 March 2023). The DMs were mapped onto Kyoto Encyclopedia of Genes and Genomes (KEGG) pathways based on database searches (http://www.genome.jp/kegg/, accessed on 7 March 2023).

### 2.7. Amino Acid Standards and Reagents

To create the typical stock solution, an analytical balance was used to precisely measure the suitable quantity of every standard substance. These substances were then dissolved in water to achieve a concentration of 1 mg mL^−1^ for each individual standard. The resulting stock solutions for each element serve as the standard stock solutions. To create the blended standard solution, working solution 1 was made by diluting suitable amounts of every standard stock solution with an appropriate solvent, such as water or a specified diluent. The concentrations of the standard solution mixture were 64,000 ng mL^−1^, 32,000 ng mL^−1^, 16,000 ng mL^−1^, 8000 ng mL^−1^, 4000 ng mL^−1^, 2000 ng mL^−1^, 1000 ng mL^−1^, 500 ng mL^−1^, 250 ng mL^−1^, 125 ng mL^−1^, 62.5 ng mL^−1^, and 31.25 ng mL^−1^.

### 2.8. Determination of Amino Acid Content

The extraction and determination methods of amino acids refer to Xiong et al. (2022) [3]. The UPLC-ESI-MS/MS technique was utilized to quantitatively detect amino acids.

The chromatographic conditions included an injection volume of 1 μL. The flow rate was 0.3 milliliters per min. The mobile phase consisted of phase A, which was an aqueous solution containing 0.2% formic acid and 10 mM formic acid ammonium, and phase B, which was an aqueous solution containing 0.2% formic acid, 90% acetonitrile, and 10 mM formic acid ammonium. The gradient elution procedure consisted of the following steps: 0 min with A/B (10:90, *v*/*v*), 2 min with A/B (10:90, *v*/*v*), 3 min with A/B (10:90, *v*/*v*), 7 min with A/B (13:87, *v*/*v*), 12.5 min with A/B (55:45, *v*/*v*), 13 min with A/B (55:45, *v*/*v*), 13.01 min with A/B (10:90, *v*/*v*), and 15 min with A/B (10:90, *v*/*v*).

The conditions for mass spectrometry were as follows: the curtain gas was set at 35 psi, and the collision-activated dissociation (CAD) parameters were set to medium. The voltage for the spray of positive ions was set at 5500 V. The voltage of the negative ion spray was −4500 V. The temperature of the ion source was 450 °C. The temperature in the column was 40 °C. The gas1 (spray gas) pressure was 55 psi. The gas2 (heating gas) pressure was 55 psi.

### 2.9. Amino Acid Data Mass Spectral Analysis

The MRM transitions for every amino acid were automatically detected and merged using the standard settings in the SCIEX OS-MQ software (version 3.1.6), created by SCIEX, an American-based company. The software facilitated the process by automatically recognizing and integrating the MRM transitions. However, manual inspection was also conducted to ensure accuracy and quality control. In Figure S2A, the TIC chromatogram of the sample displays the overall ion intensity observed throughout the duration. The horizontal axis indicates the time it takes for metabolite detection (RT), while the vertical axis indicates the intensity of ions in counts per second (cps). The extracted ion chromatogram of the negative ion detection sample is shown in Figure S2B. It exhibits the separation and peak shape of various substances. The horizontal axis indicates the time it takes for metabolite detection (RT), while the vertical axis indicates the intensity of ions in counts per second (cps). Figure S2C illustrates the extracted ion chromatogram of the sample for detecting positive ions. It also demonstrates high separation and well-defined peak shapes. The horizontal axis indicates the time it takes for metabolite detection (RT), while the vertical axis indicates the intensity of ions in counts per second (cps). Based on Table S1, the linear regression examination of the standard curves for various metabolites reveals that the coefficient of determination (R2) exceeds 0.99 for every metabolite. This suggests a strong correlation between the levels of the metabolites and the corresponding peak sizes. The high R2 values suggest a strong linear relationship, indicating good linearity in the standard curves. The overlaid chromatograms of the total ion current (TIC) obtained from analyzing various QC samples are shown in Figure S2D,E. The significant similarity of the curves in the TIC chromatogram for metabolite detection suggests excellent signal consistency in the mass spectrometry and liquid chromatography systems across various time intervals. Table S1 includes details about the retention time, limit of detection (LOD), and limit of quantitation (LOQ) for every standard compound. Table S1 includes the relative standard deviation (RSD) for every metabolite, with all RSD values being below 14%. This implies that the technique and evaluation system are steady and trustworthy, rendering it appropriate for the quantitative examination of the samples.

### 2.10. Statistical Analysis

The biochemical data were organized and plotted using Adobe Illustrator CS6 and WPS2021. Correlation analysis and significance analysis (Tukey’s test) were conducted using SPSS 18.0 software.

## 3. Results

### 3.1. Analysis of Multiple Variables

**Figure 1:**
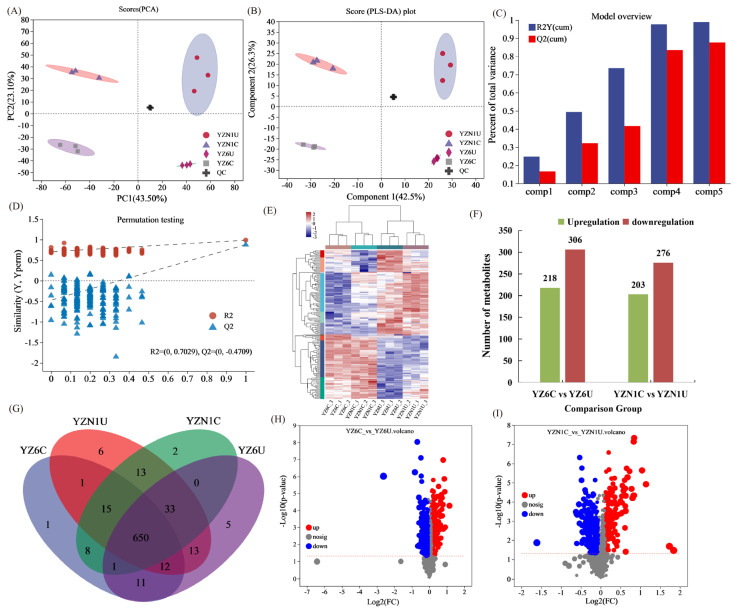
Overview of grain metabolite information. (**A**) PCA score plot, (**B**) PLS-DA score plot, (**C**) overview of the PLS-DA model, (**D**) PLS-DA permutation testing, (**E**) metabolite clustering heatmap, (**F**) up- and downregulation of differential metabolites (DMs), (**G**) metabolite Venn diagram, (**H**) volcano plot of YZ6C vs. YZ6U comparison, (**I**) volcano plot of YZN1C and YZN1U comparison. YZN1U, YZN1 uncooked; YZN1C, YZN1 cooked; YZ6U, YZ6 uncooked; YZ6C, YZ6 cooked.

This study involved conducting differential comparisons of the primary metabolite constituents of twelve grain samples. To determine the variability and patterns in the data, the determination of the contribution rates of PC1 and PC2 was carried out in principal component analysis (PCA). From the PCA score plot, it can be observed that PC1 accounts for 43.5% of the total variance, while PC2 accounts for 23.1%. The combined contribution of these two principal components amounts to 56.6% of the total variance (Figure 1A). This study conducted differential comparisons of the primary metabolite constituents of twelve grain samples. The significant differences observed among the four samples in the PCA score plot indicate distinct variations between these samples. This suggests that there are notable differences in the major metabolite components among the analyzed rice grain samples. Moreover, the data underwent a partial least squares discriminant analysis (PLS-DA). Figure 1B displays the findings of the PLS-DA analysis, indicating that component 1 accounts for 42.5% of the variability, whereas component 2 accounts for 26.3% of the variability. These results indicate that there is significant separation between the samples, which is consistent with the findings from the PCA. The modeling and predictive abilities of the PLS-DA model can be evaluated using R2Y and Q2 values. The cumulative values of R2Y and Q2 indicate the stability and reliability of the model. Figure 1C illustrates that a greater combined R2Y and Q2 indicates a model that is more dependable and consistent. From the trend of the regression line and the decrease in R2 and Q2, the overall trend shows an upward slope (Figure 1D). This suggests that the permutation test conducted for this experiment was effective, and the model shows no signs of overfitting. The heatmap of metabolite hierarchical clustering demonstrates the remarkable consistency among the replicates of the samples (Figure 1E). In the YZ6C and YZ6U comparison groups, 218 differentially upregulated metabolites and 306 differentially downregulated metabolites were identified (Figure 1F; Table S2). In the YZN1C and YZN1U comparison groups, 203 differentially upregulated metabolites and 276 differentially downregulated metabolites were observed. Notably, there was an increased quantity of metabolites that were downregulated following the cooking procedure of purple rice (Figure 1F; Table S3). Distinct differences in the composition of metabolites were observed in each comparison group following the cooking of purple rice, as indicated by the presence of specific metabolite components (Figure 1G). Additionally, volcano plots were generated to visually represent the DMs in the YZ6C and YZ6U comparison (Figure 1H) and YZN1C and YZN1U comparison (Figure 1I). These volcano plots provide an intuitive display of upregulated and downregulated metabolites.

### 3.2. Statistical Chart Analysis of Compounds

The study utilized pie charts to visually represent the distribution of metabolites within specific hierarchical structures (classes) of the Human Metabolome Database (HMDB). Among YZ6C and YZ6U, the highest percentage of metabolites was attributed to organooxygen compounds, accounting for 14.63%. Flavonoids accounted for 13.21%, followed by carboxylic acids and derivatives at 10.16%. Further analysis of flavonoids in the subclasses revealed that flavonoid glycosides accounted for 9.15%, flavones accounted for 2.03%, isoflavonoid O-glycosides accounted for 1.02%, and flavans accounted for 1.63%. Importantly, the collective ratio of organooxygen substances and flavonoids surpassed a quarter of all metabolites, suggesting their greater prevalence in the YZ6C and YZ6U comparison group (Figure S3A). Flavonoids comprised the highest percentage, making up 14.48% of the metabolites, when comparing YZN1C and YZN1U. Organooxygen compounds accounted for 13.36%. Further analysis of flavonoids in the subclasses revealed that flavonoid glycosides accounted for 9.35%, flavones accounted for 1.34%, o-methylated flavonoids accounted for 1.56%, and flavans accounted for 1.78% (Figure S3B). The remaining categories had relatively smaller proportions. Similar to the YZ6C and YZ6U comparison, the combined proportion of organooxygen compounds and flavonoids in the YZN1C and YZN1U comparison group still exceeded one-fourth of the total metabolites, indicating their higher abundance (Figure S3A,B). This suggests that these two classes of metabolites are also relatively abundant and potentially play significant roles in the observed metabolic differences.

### 3.3. KEGG Analysis

**Figure 2:**
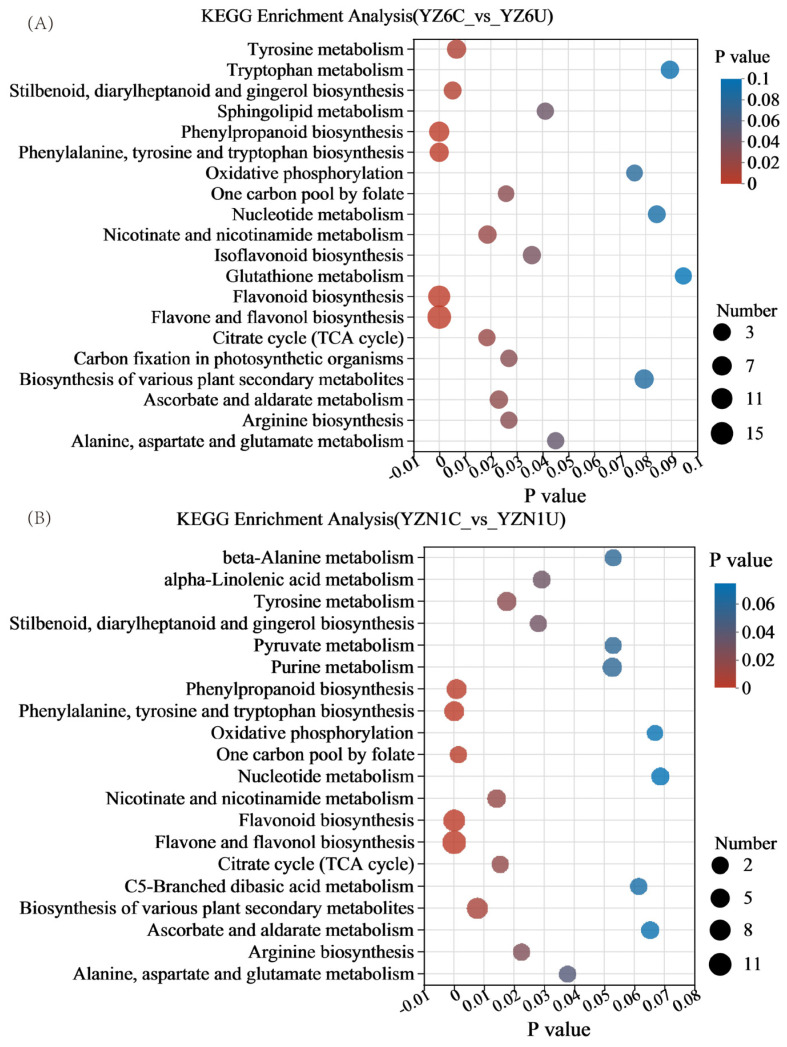
KEGG enrichment analysis plot. (**A**) KEGG Enrichment Analysis (YZ6C_vs_YZ6U), (**B**) KEGG Enrichment Analysis (YZ1C_vs_YZ1U). The x−axis represents the significance *p* value of enrichment, where a smaller *p* value indicates greater statistical significance. Typically, a *p* value below 0.05 is considered significantly enriched for a particular function. The y−axis represents KEGG pathways. The size of the bubbles in the plot indicates the number of enriched compounds within the metabolic set for that pathway. YZN1U, YZN1 uncooked; YZN1C, YZN1 cooked; YZ6U, YZ6 uncooked; YZ6C, YZ6 cooked.

The KEGG enrichment analysis revealed significant pathway involvement of DMs between the YZ6C and YZ6U comparison groups (Figure 2A). The pathways that were enhanced with statistical significance (*p* < 0.05) consisted of tyrosine breakdown; tryptophan breakdown; production of phenylpropanoid compounds; production of phenylalanine, tyrosine, and tryptophan; production of isoflavonoids; metabolism of glutathione; production of flavonoids; production of flavones and flavonols; tricarboxylic acid cycle (TCA cycle); production of arginine; and metabolism of alanine, aspartate, and glutamate. The analysis of enrichment in the pathways of flavonoid biosynthesis and flavone and flavonol biosynthesis indicated the most significant enrichment of metabolites in these pathways. This suggests that the metabolic differences observed in these pathways are primarily associated with flavonoid metabolites. The direct messages exchanged between the YZN1C and YZN1U comparison groups were discovered to be engaged in various metabolic pathways, such as beta-alanine processing; phenylpropanoid creation; phenylalanine, tyrosine, and tryptophan creation; flavonoid creation; flavone and flavonol creation; TCA cycle; arginine creation; and alanine, aspartate, and glutamate metabolism (*p* < 0.05) (Figure 2B). The flavonoid biosynthesis and flavone and flavonol biosynthesis pathways exhibited the greatest enrichment of metabolites among these pathways, suggesting that the differentially regulated metabolites predominantly pertain to the flavonoid category.

### 3.4. Analysis of Flavonoids, Total Antioxidant Capacity, and Related Nutrient Content

In this research, a comparative analysis was performed on the levels of flavonoids, total antioxidant capacity (TAC), and other nutritional constituents in 12 different grain seed samples (Table 1). Significant differences were observed in the flavonoid content, TAC, OPC, and TP between cooked and uncooked purple rice samples. YZN1C exhibited lower levels of flavonoids, OPC, TP, DPPH, FRAP, and ABTS by 43.2%, 78.9%, 46.5%, 45.6%, 15.4%, and 48.5%, respectively, compared to YZN1U. Similarly, YZ6C showed lower levels of flavonoids, OPC, TP, DPPH, FRAP, and ABTS by 20%, 70.1%, 37.9%, 43.8%, 41.7%, and 21.9%, respectively, compared to YZ6U. During the cooking process of purple rice, there was a significant decrease (*p* < 0.05) in the total antioxidant capacity, TP, and flavonoid content. Compared to YZN1U, YZN1C showed increased levels of most of its major nutritional components, except for cellulose. Among them, crude protein exhibited the highest increase, reaching 12.2%. YZ6C cellulose, ash, crude protein, and dietary fiber showed higher levels than YZ6U, whereas the major nutritional components, total starch and total sugar, exhibited an increase. Among them, the increase in TS was the highest, reaching 24.2%. YZN1U exhibited higher TAC and flavonoid content than YZ6U. YZN1C had a higher TAC than YZ6C but a lower total flavonoid content. Among the major nutritional components, except for TS and cellulose, YZN1C had higher levels of the remaining components than YZ6C.

### 3.5. Differential Analysis of Mineral Element Content

This study conducted a mineral element analysis of 12 grain samples (Table 2). Both types of purple rice showed varying degrees of nutrient element loss during the cooking process. The mineral element content in YZN1C was lower than that in YZN1U. Compared to YZN1U, the levels of P, K, Na, Fe, Mn, Zn, Cu, Ca, and Mg in YZN1C were reduced by 2.2%, 60%, 28%, 31.7%, 50.5%, 45.2%, 47%, 21%, and 59.7%, respectively. The K content showed the largest decrease, while the P content showed the smallest decrease. This suggests that the culinary procedure greatly affects the nutritional component K while having a comparatively lesser effect on P. YZ6C exhibited a lower mineral element content compared to YZ6U. Compared to YZ6U, YZ6C exhibited decreases in the levels of P, K, Na, Fe, Mn, Zn, Cu, Ca, and Mg by 4.2%, 56.6%, 25.8%, 25.1%, 52.7%, 48.9%, 35.2%, 33.3%, and 50.3%, respectively. Similarly, K exhibited the largest decrease, while P showed the smallest decrease. The cooking process has a notable effect on K, leading to a considerable reduction, while the P content remains relatively unchanged throughout the cooking procedure.

### 3.6. Differential Analysis of Amino Acid Content

This study analyzed a total of 25 amino acids and observed that the two purple rice varieties exhibited varying degrees of changes in amino acid content during the cooking process (Table 3). In YZN1C, the contents of Asn, Asp, Glu, Gln, Ile, Phe, Tyr, and Val were significantly lower than those in YZN1U, with decreases of 10.4%, 8.7%, 30.6%, 47.9%, 15.9%, 13.6%, 19.2%, and 24.1%, respectively. Among them, the difference in Gln content was the most significant, indicating a significant loss of Gln during the cooking process. On the other hand, the contents of b-Ala, GABA, Ala, Arg, Cys, His, Lys, Ser, and Tau increased compared to YZN1U. In YZ6C, the contents of Asn, Glu, Gln, and Met were significantly lower than those in YZ6U, with decreases of 5.2%, 41.7%, 41.7%, and 24.3%, respectively. Among them, the difference in Gln and Glu content was the most pronounced, indicating a significant loss of Glu and Gln during the cooking process. On the other hand, the contents of 4-Hpro, b-Ala, GABA, Ala, Arg, Asp, Gly, His, Ile, Leu, Lys, Orn, Phe, Ser, Thr, Val, and Tau increased compared to YZ6U. In YZ6U, except for Arg, Asn, Glu, His, Met, Pro, and Try, the content of other amino acids was lower than that in YZN1U. This indicates that the YZN1 variety has a richer amino acid content. In YZ6C, except for GABA, Arg, Asn, Asp, Glu, His, Pro, and Try, the content of other amino acids was lower than that in YZN1C. This suggests that YZN1 has higher nutritional value than YZ6 during the cooking process of purple rice.

### 3.7. Flavonoid, Total Antioxidant Capacity, Nutrient, and Metabolite Correlation Analysis

**Figure 3:**
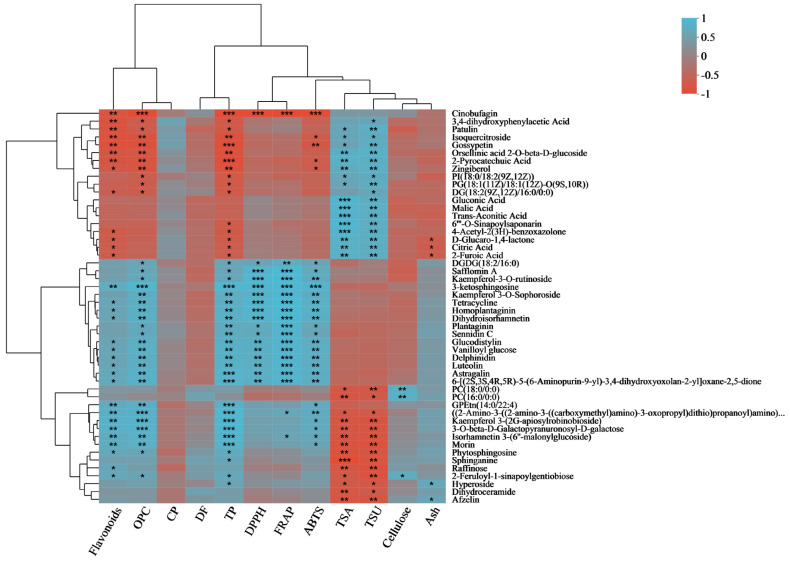
Correlation analysis of flavonoids, total antioxidant capacity, and related nutritional components with DMs. The right side shows the names of DMs, and the bottom indicates flavonoids, total antioxidant capacity, and related nutritional components. Each grid represents the correlation between the two attributes, and different colors represent the sizes of the correlation coefficients between the attributes. * *p* < 0.05; ** *p* < 0.01; *** *p* < 0.001.

To comprehend the connection between different biochemical markers and metabolites, we performed a correlation analysis involving flavonoids, OPC, CP, DF, TP, DPPH, FRAP, ABTS, TSA, TSU, cellulose, ash markers, and metabolites (Figure 3). Isoquercitroside demonstrated a notable positive association with TSA (total soluble anthocyanins) and TSU (total soluble sugars), whereas it displayed a significant negative correlation with ABTS, TP, flavonoids, and OPC. The TP, DPPH, FRAP, and ABTS radical scavenging activities were significantly correlated with kamepferol 3-O-sophoroside, kamepferol 3-O-rutinoside, dihydroisorhamnetin, and luteolin. Dihydroisorhamnetin, delphinidin, and luteolin, among others, displayed a noteworthy association with flavonoids and OPC, indicating a positive correlation. TSA and TSU had a strong negative correlation with kaempferol 3-(2G-apiosylroboinobioside), isorhamnetin 3-(6″-malonylglucoside), and morin, whereas they displayed a notable positive correlation with TP, flavonoids, and OPC. Isorhamnetin 3-(6″-malonylglucoside) also showed a significant positive correlation with FRAP. Hyperoside demonstrated a notable positive association with TP and Ash, whereas it displayed a significant negative correlation with TSA and TSU.

### 3.8. Mineral Element and Metabolite Correlation Analysis

**Figure 4:**
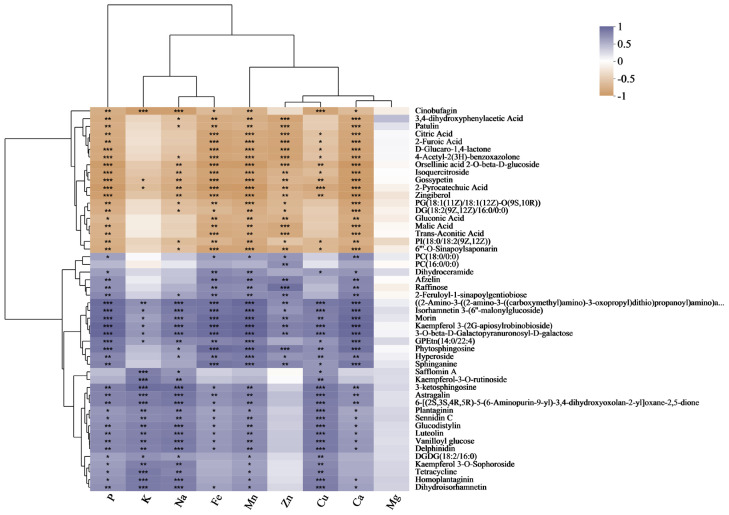
Correlation analysis of P, K, Na, Fe, Mn, Zn, Cu, Ca, and Mg with DMs. The right side shows the names of DMs, and the bottom indicates mineral elements. Each grid represents the correlation between the two attributes, and different colors represent the sizes of the correlation coefficients between the attributes. * *p* < 0.05; ** *p* < 0.01; *** *p* < 0.001.

To understand the relationship between metabolites and mineral elements, we conducted a correlation analysis between P, K, Na, Fe, Mn, Zn, Cu, Ca, and Mg indicators and metabolites (Figure 4). Isoquercitroside showed a significant negative correlation with P, Na, Fe, Mn, Zn, Cu, and Ca. Isorhamnetin 3-(6″-malonylglucoside), morin, kaempferol 3-(2G-apiosylrobinobioside), and hyperoside all exhibited a significant positive correlation with P, Na, Fe, Mn, Zn, Cu, and Ca. Among them, isorhamnetin 3-(6″-malonylglucoside), morin, and kaempferol 3-(2G-apiosylrobinobioside) also showed a significant positive correlation with K. Kaempferol-3-O-rutinoside showed a significant positive correlation with K, Na, and Cu. Kaempferol 3-O-sophoroside exhibited a significant positive correlation with P, K, Na, Mn, and Cu. Astragalin, luteolin, delphinidin, and dihydroisorhamnetin all showed a significant positive correlation with P, K, Na, Fe, Mn, Cu, and Ca.

### 3.9. Amino Acid and Metabolite Correlation Analysis

**Figure 5:**
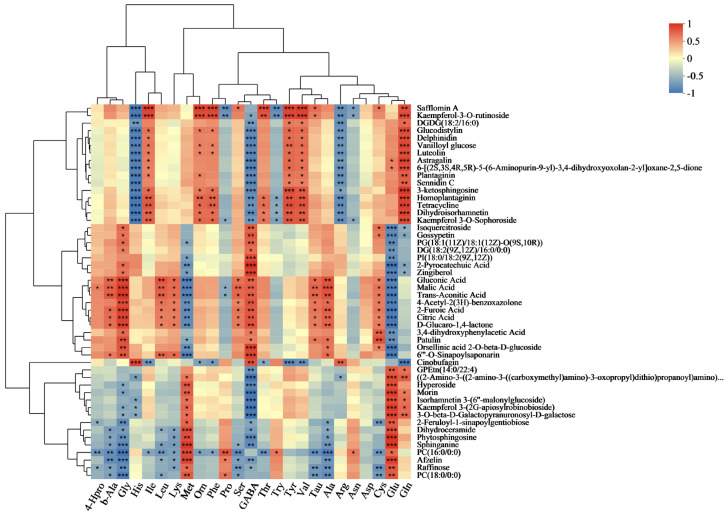
Correlation analysis of amino acids with DM. The right side shows the names of DMs, and the bottom indicates amino acids. Each grid represents the correlation between the two attributes, and different colors represent the sizes of the correlation coefficients between the attributes. * *p* < 0.05; ** *p* < 0.01; *** *p* < 0.001.

To comprehend the connection between metabolites and amino acids, we performed a correlation analysis involving the following indicators: 4-Hpro, b-Ala, Gly, His, He, Leu, Lys, Met, Orn, Phe, Pro, Ser, GABA, Thr, Try, Tyr, Val, Tau, Ala, Arg, Asn, Asp, Cys, Glu, and Gln, along with the metabolites (Figure 5). A significant inverse relationship was observed between kaempferol-3-O-rutinoside and Gly, Pro, GABA, Try, Arg, and Asn. A strong positive correlation was observed with His, Orn, Phe, Thr, Tyr, Val, Tau, and Gln. Both delphinidin and luteolin exhibited a notable positive association with Ile, Tyr, Val, and Gln while demonstrating a significant negative association with histidine, GABA, and arginine. There was a strong positive correlation between dihydroisorhamnetin and kaempferol 3-O-sophoroside and Ile, Orn, Phe, Thr, Tyr, Val, and Gln, while a notable negative correlation was observed with His, GABA, Try, and Arg. Isoquercitroside showed a strong positive association with Gly, GABA, and Cys, while displaying a notable negative association with Glu and Gln. A significant positive correlation was observed between hyperoside, morin, isorhamnetin 3-(6″-malonylglucoside), and kaempferol 3-(2G-apiosylrobinobioside). Additionally, kaempferol 3-(2G-apiosylrobinobioside) and isorhamnetin 3-(6″-malonylglucoside) showed significant positive correlations with Gln and significant negative correlations with His.

### 3.10. Receiver Operating Characteristic (ROC) Analysis

**Figure 6:**
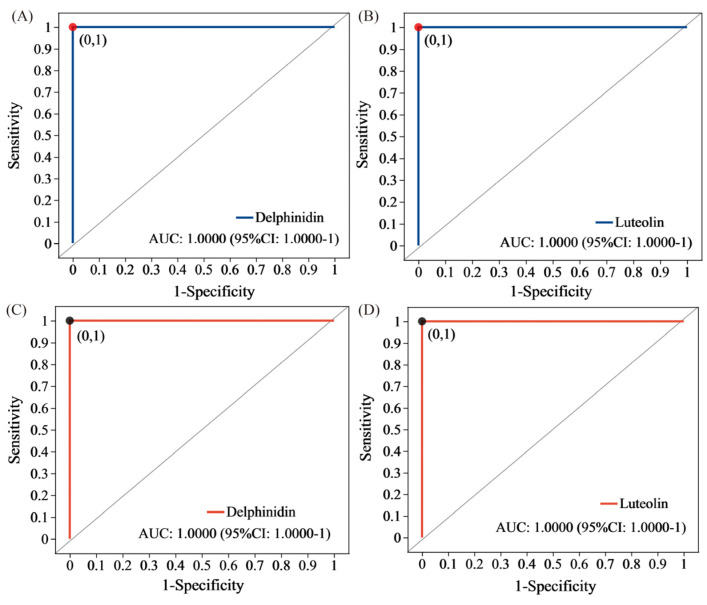
ROC analysis. (**A**) ROC analysis of delphinidin between YZ6U and YZ6C, (**B**) ROC analysis of luteolin between YZ6U and YZ6C, (**C**) ROC analysis of delphinidin between YZN1U and YZN1C, (**D**) ROC analysis of luteolin between YZN1U and YZN1C. YZN1U, YZN1 uncooked; YZN1C, YZN1 cooked; YZ6U, YZ6 uncooked; YZ6C, YZ6 cooked.

The significance of intergroup differences and the similarity between groups in a grouped sample can be evaluated using ROC analysis. Moreover, sample partitioning can generate sample groups that can be used for linear discrimination and classification modeling, which can be accomplished by utilizing supervised learning methods. This approach also enables the identification of key metabolite variables (biomarkers) that contribute significantly to intergroup discrimination (Figure 6). The areas under the curve (AUCs) of delphinidin and luteolin between YZ6U and YZ6C were 1 and 1, respectively (Figure 6A,B). The AUCs of delphinidin and luteolin between YZN1U and YZN1C were 1 and 1, respectively (Figure 6C,D), where the AUC is greater than 0.5, and a higher value approaching 1 indicates better diagnostic performance.

## 4. Discussion

Consumers of rice can benefit from the bioactive antioxidants found in purple rice, which is abundant in polyphenols, flavonoids, anthocyanins, and various other antioxidative nutrients [21,22]. Cooking, which involves high-temperature continuous heating, not only sterilizes and disinfects the rice but also alters the nutritional composition, making it more easily digestible and absorbable [23]. The study found that the levels of flavonoids, OPC, TP, and TAC showed a significant decrease in both varieties of purple rice after cooking (Table 1). Delphinidin and luteolin exhibited strong positive correlations with flavonoids, OPC, TP, DPPH, FRAP, ABTS, P, Ka, Na, Fe, Mn, Cu, and Ca, as revealed by the correlation analysis of differential metabolites, flavonoids, total antioxidant capacity, mineral elements, and amino acid indicators following the steaming process of two types of purple rice. Significant positive correlations were observed between delphinidin and luteolin and He, Try, Tyr, and Gln, whereas significant negative correlations were found with His, GABA, and Arg. The KEGG pathway analysis revealed that the comparative groups YZN1C and YZN1U, and YZ6C and YZ6U exhibited significant enrichment of the pathways primarily associated with the biosynthesis of flavonoids, flavones and flavonols (Table 2). This suggests that the distinct metabolites in these two comparison groups are mainly linked to flavonoid substances (Figure S3A,B). The compound statistics of the two comparative groups further corroborate the observation. In the comparison of YZ6C and YZ6U, flavonoids accounted for 13.21% (Figure S3A). In the comparative group of YZN1C and YZN1U, flavonoids had the highest proportion, accounting for 14.48% (Figure S3B). There was a strong positive correlation between delphinidin, luteolin, and flavonoids. The amino acid content of YZN1C was notably lower than that of YZN1U after steaming, as indicated by the decrease in the levels of Asn, Asp, Glu, Gln, Ile, Phe, Tyr, and Val. Among these, Gln showed the highest loss in content after steaming of YZN1 purple rice. YZ6C exhibited significantly reduced levels of Asn, Glu, Gln, and Met compared to YZ6U. In addition, Gln experienced a substantial loss in content after steaming of YZ6 purple rice. This suggests that Gln is the primary amino acid that is depleted when purple rice is steamed. After analyzing the correlation between different metabolites and amino acids following the steaming process of the two types of purple rice, it was discovered that Gln exhibited a notable positive correlation with delphinidin and luteolin.

Flavonoid compounds have been shown to effectively scavenge free radicals, making them highly beneficial in terms of antioxidant and anti-aging effects [24,25]. Anthocyanins are primarily used for food coloring purposes but can also find applications in dyes, pharmaceuticals, cosmetics, and other fields [26,27]. According to the findings of this research, the levels of primary anthocyanins in YZN1U were considerably greater than those in YZN1U, YZ6U, and YZ6C, exhibiting variances of 373.21%, 108.66%, and 597.37%, respectively (Table 1). Fe is an essential element in the creation of hemoglobin, and a lack of this mineral in the human body can result in inadequate production of red blood cells and the development of anemia caused by iron deficiency [28,29]. Fe exhibits a notable positive correlation with delphinidin and luteolin. The Fe content varies after steaming the two types of purple rice. Notably, even after steaming, purple rice still contains higher levels of mineral elements, including Fe, compared to regular white rice [6]. Hence, the creation of pigmented rice types with higher levels of antioxidants and enhanced resistance to cooking can offer a convenient way for individuals to obtain enough antioxidant compounds in their diet. This can enhance the quality of human life and meet the demands for a healthy lifestyle. By analyzing the correlation between biochemical indicators and metabolites, this study discovered a robust association among delphinidin, luteolin, flavonoids, TAC, mineral elements, and amino acid indicators. Flavonoids, a subclass of phenolic compounds, including anthocyanins [30], are abundant natural sources of antioxidants in the human diet. They can effectively eliminate free radicals and help prevent many diseases [31]. Delphinidin is a common anthocyanin compound that has shown promising effects in cancer prevention, antitumor activities, immune enhancement, and cardiovascular disease prevention [32,33]. Luteolin, a flavonoid compound, can alter the breakdown of dietary starch, decrease food consumption, regulate antioxidant enzyme function to enhance oxidative stress management, safeguard pancreatic islet function, reverse macrophage polarization to alleviate insulin resistance caused by a high-fat diet, and provide protection for insulin secretion by β-cells to counteract the progression of diabetes [34,35]. Metabolic markers have been widely used in food quality, food traceability, and safety research. Through the identification of metabolic markers, we can evaluate food quality and safety [36]. Significant positive correlations were observed between delphinidin and luteolin based on the correlation analysis of DMs with flavonoids, OPC, TP, DPPH, FRAP, ABTS, P, Ka, Na, Fe, Mn, Cu, and Ca. Delphinidin and luteolin exhibit notable positive associations with the amino acids He, Try, Tyr, and Gln, while displaying significant negative associations with His, GABA, and Arg. Therefore, delphinidin and luteolin can serve as indicative markers for the loss of nutritional compounds after steaming of purple rice. After performing a ROC analysis, it was discovered that delphinidin and luteolin exhibited a perfect AUC value of 1 in the comparative groups of the two types of purple rice, both prior to and following the steaming process. This indicates excellent diagnostic performance, further confirming that delphinidin and luteolin are reliable biomarkers for the loss of nutritional compounds after steaming of purple rice. Hence, this research offers a significant conceptual foundation for developing novel rice cultivars that possess elevated nutritional value and promote well-being. Additionally, this study contributes to the efficient utilization of the nutrient composition in cooked colored rice and enriches the dietary structure of humans by incorporating such rice varieties.

## 5. Conclusions

The levels of flavonoids, OPC, TP, K, Na, Fe, Mn, Zn, Cu, Ca, and Mg were significantly reduced after preparing a dish with purple rice. The use of YZN1 in cooking resulted in a reduction in the levels of 14 amino acids, specifically Asn, Asp, Glu, Gln, Ile, Leu, Met, Orn, Phe, Pro, Thr, Try, Tyr, and Val. The usage of YZ6 in the cooking process led to a reduction in the levels of seven amino acids, specifically Asn, Glu, Gln, Met, Pro, Try, and Tyr. Two purple rice samples lost the most Gln after cooking. The primary involvement of the DMs in the production of the two purple rice varieties is in the pathways of flavonoid biosynthesis and flavone and flavonol biosynthesis. The DMs primarily consist of flavonoids. Flavonoids made up the highest percentage, 13.21%, in the YZ6C and YZ6U comparison groups. In both the YZN1C and YZN1U comparison groups, the percentage of flavonoids was also the greatest, reaching 14.48%. Flavonoids, OPC, TP, DPPH, FRAP, ABTS, P, Ka, Na, Fe, Mn, Cu, Ca, He, Try, Tyr, Gln, His, GABA, and Arg strongly correlated with delphinidin and luteolin contents. Delphinidin and luteolin can be used as key chemicals for nutrient loss after purple rice cooking. Through ROC analysis, the AUC values of delphinidin and luteolin were both 1, indicating a good diagnostic effect. This further confirms that delphinidin and luteolin are biomarkers of nutrient loss after purple rice cooking. This research offers a scientific foundation for the development of novel rice cultivars with enhanced nutritional content and the utilization of the nutritional value of cooked colored rice. In the future, we should expand the sample size and conduct experiments to explore the impact of cooking time on the quality of purple rice and other foods. Clarifying metabolic markers not only plays an important role in identifying food quality but also has enormous application value in tracing food adulteration.

## Figures and Tables

**Table 1:** Differences in flavonoids, total antioxidant capacity, and related nutritional component content between uncooked and cooked purple rice.

Name	YZN1U	YZN1C	YZ6U	YZ6C
Flavonoids (mg g^−1^ DW)	9.62 a	5.46 c	7.54 b	6.03 c
OPC (mg g^−1^ DW)	2.65 a	0.56 c	1.27 b	0.38 c
TP (mg g^−1^ DW)	6.45 a	3.45 c	4.91 b	3.05 c
DPPH (μmol Trolox g^−1^ DW)	10.61 a	5.77 b	3.22 c	1.81 d
FRAP (μmol Trolox g^−1^ DW)	10.58 a	8.95 b	7.27 c	4.24 d
ABTS (μmol Trolox g^−1^ DW)	44.91 a	23.12 bc	25.53 b	19.94 c
Total starch (mg g^−1^ DW)	521.48 a	556.66 a	453.69 b	563.58 a
Total sugar (mg g^−1^ DW)	156.16 bc	176.20 a	147.34 c	169.76 ab
Cellulose (mg g^−1^ DW)	31.87 ab	29.97 b	33.93 a	33.88 a
Ash (%)	1.30% ab	1.35% ab	1.54% a	1.17% b
Crude protein (g kg^−1^)	78.81 a	88.44 a	82.70 a	79.04 a
Dietary fiber (g 100 g^−1^)	38.41 a	39.33 a	38.37 a	37.01 a

OPC: oligomeric proanthocyanidin; TP: total phenols; ABTS: 2,2′-azino-bis(3-ethylbenzothiazoline-6-sulfonic acid; DPPH: 2,2-diphenyl-1-picrylhydrazyl; FRAP: ferric ion reducing antioxidant power. YZN1U, YZN1 uncooked; YZN1C, YZN1 cooked; YZ6U, YZ6 uncooked; YZ6C, YZ6 cooked. Lower-case letters represent the significance of the *P* value at the 0.05 level.

**Table 2:** Differences in mineral element content between uncooked and cooked purple rice.

Name	YZN1U	YZN1C	YZ6U	YZ6C
P (g kg^−1^)	3.25 a	3.18 a	3.60 a	3.45 a
K (g kg^−1^)	1.95 a	0.78 c	1.75 b	0.76 c
Na (mg kg^−1^)	263.19 a	189.51 b	189.69 b	140.73 c
Fe (mg kg^−1^)	141.79 a	96.88 c	114.73 b	85.95 d
Mn (mg kg^−1^)	25.54 a	12.65 b	27.22 a	12.87 b
Zn (mg kg^−1^)	20.94 a	11.47 b	20.88 a	10.68 b
Cu (mg kg^−1^)	3.28 ab	1.74 c	3.98 a	2.58 b
Ca (mg kg^−1^)	336.44 a	265.95 b	313.91 a	209.51 c
Mg (mg kg^−1^)	1094.14 a	441.28 c	1065.71 a	529.72 b

Lower-case letters represent the significance of the *p* value at the 0.05 level.

**Table 3:** Differences in amino acid content between uncooked and cooked purple rice.

Name	YZN1U	YZN1C	YZ6U	YZ6C
4-Hpro	910.97 a	968.78 a	780.34 a	924.66 a
b-Ala	4661.50 a	4758.96 a	2580.01 c	3869.98 b
GABA	11,167.20 c	42,466.62 b	8245.45 c	63,503.05 a
Ala	186,191.47 b	209,312.39 a	139,039.34 c	169,689.40 b
Arg	265,796.44 b	280,304.41 b	294,406.48 ab	325,229.20 a
Asn	268,552.20 bc	257,955.75 c	331,560.66 a	314,272.70 ab
Asp	434,741.15 a	396,841.79 ab	327,149.06 b	399,860.46 ab
Cys	348.48 b	1331.48 a	0.00	0.00
Glu	649,528.57 b	450,483.26 c	777,920.98 a	453,643.33 c
Gln	152,509.77 a	79,477.97 b	79,592.49 b	46,372.64 c
Gly	42,381.16 b	51,521.92 a	27,391.61 c	41,473.36 b
His	17,140.27 c	19,810.90 bc	22,550.80 b	32,258.33 a
Ile	17,265.17 a	14,516.98 b	7269.94 b	7827.60 b
Leu	61,892.60 a	59,930.33 a	41,562.00 b	56,443.22 a
Lys	64,504.26 a	67,700.66 a	51,797.24 b	62,056.12 ab
Met	1634.11 b	1531.42 b	2000.62 a	1515.12 b
Orn	6893.36 a	6845.44 a	3232.35 b	3350.01 b
Phe	37,691.70 a	32,574.72 b	17,537.64 c	19,916.29 c
Pro	17,646.80 b	14,969.46 b	27,222.65 a	25,333.40 a
Ser	132,369.94 a	139,742.35 a	103,033.81 b	116,182.70 b
Thr	28,914.74 a	27,433.44 a	15,646.30 c	18,450.26 b
Try	34,376.07 b	32,810.18 b	50,102.78 a	48,492.18 a
Tyr	22,342.79 a	18,029.16 b	14,341.00 c	13,969.16 c
Val	34,089.36 a	25,858.97 b	13,524.16 c	13,834.82 c
Tau	8079.56 b	9247.74 a	5849.12 c	6582.99 c

Values are expressed as (ng g^−1^). 4-Hpro: 4-hydroxyproline; b-Ala: beta-alanine; GABA: gamma-aminobutyric acid; Ala: alanine; Arg: arginine; Asn: asparagine; Asp: aspartic acid; Cys: cysteine; Glu: glutamic acid; Gln: glutamine; Gly: glycine; His: histidine; Ile: isoleucine; Leu: leucine; Lys: lysine; Met: methionine; Orn: ornithine; Phe: phenylalanine; Pro: proline; Ser: serine; Thr: threonine; Try: tryptophan; Tyr: tyrosine; Val: valine; Tau, taurine. YZN1U, YZN1 uncooked; YZN1C, YZN1 cooked; YZ6U, YZ6 uncooked; YZ6C, YZ6 cooked. Lower-case letters represent the significance of the *P* value at the 0.05 level.

## Data Availability

The data presented in this study are available on request from the corresponding author. The data are not publicly available due to restrictions privacy.

## Supplementary Materials

The following supporting information can be downloaded at https://www.mdpi.com/article/10.3390/metabo13091018/s1, Figure S1. Two purple rice pictures. Figure S2: Ion current diagram. (A) Total ion current diagram (standard), (B) extraction of negative ion current, (C) extraction of positive ion current diagram, (D) total ion current diagram (QC sample superposition). Figure S3: Statistical map of compounds. The name and percentage of the metabolites in the selected HMDB hierarchy (class) are displayed in a high to low order, depending on the number of metabolites. The different colors in each pie plot in the figure represent a different HMDB classification, the area of which represents the relative proportion of the metabolites in that classification. (A) YZ6C and YZ6U, (B) YZN1C and YZN1U; Table S1: The linear equations for the flavonoids and phenolic compounds. Table S2: Summary chart comparing metabolite details for YZ6C and YZ6U; Table S3: Summary chart displaying metabolite details for YZN1C and YZN1U.

## Abbreviations

TPC: total phenolic content; TFC: total flavonoid content; OPC: oligomeric proanthocyanidin; TP: total phenols; ABTS method: 2,2′-azino-bis(3-ethylbenzothiazoline-6-sulfonic acid; DPPH method: 2,2-diphenyl-1-picrylhydrazyl; FRAP method: ferric ion reducing antioxidant power method; TSA: total starch; TSU: total sugar; ICP-MS/AES: inductively coupled plasma-mass spectrometry/atomic emission spectrometry; QC: quality control; UHPLC-MS/MS: ultrahigh-performance liquid chromatography-mass spectrometry; ESI: electrospray ionization; ISVF: ion-spray voltage floating; DDA: data-dependent acquisition; RT: retention time; HMDB: Human Metabolome Database; VIP: variable importance in the projection; DMs: differentially expressed metabolites; KEGG: Kyoto Encyclopedia of Genes and Genomes; GEPs: gradient elution procedures; CAD: collision-activated dissociation; TIC: total ion current; cps, counts per second; XIC: extracted ion chromatogram; LOD: limit of detection; LOQ: limit of quantitation; RSD: relative standard deviation; PCA: principal component analysis; PLS-DA: partial least squares discriminant analysis; TCA cycle: tricarboxylic acid cycle; TAC: total antioxidant capacity; TP: total phenolics; DF: dietary fiber; CP: crude protein; 4-Hpro: 4-hydroxyproline; b-Ala: beta-alanine; GABA: gamma-aminobutyric acid; Ala: alanine; Arg: arginine; Asn: asparagine; Asp: aspartic; Cys: cysteine; Glu: glutamic acid; Gln: glutamine; Gly: glycine; His: histidine; Ile: isoleucine; Leu: leucine; Lys: lysine; Met: methionine; Orn: ornithine; Phe: phenylalanine; Pro: proline; Ser: serine; Thr: threonine; Try: tryptophan; Tyr: tyrosine; Val: valine; Tau: taurine; ROC: receiver operating characteristic; AUC: area under the curve.
