# Supplementary material for: Comparison of Metabolites and Main Nutritional Components between Uncooked and Cooked Purple Rice
Source: Metabolites. 2023 Sep 15;13(9):1018. doi: 10.3390/metabo13091018 (PMC10536460; doi:10.3390/metabo13091018)
Supplement: Supplementary file 1 [file metabolites-13-01018-s001.zip › Figure S2.pdf]

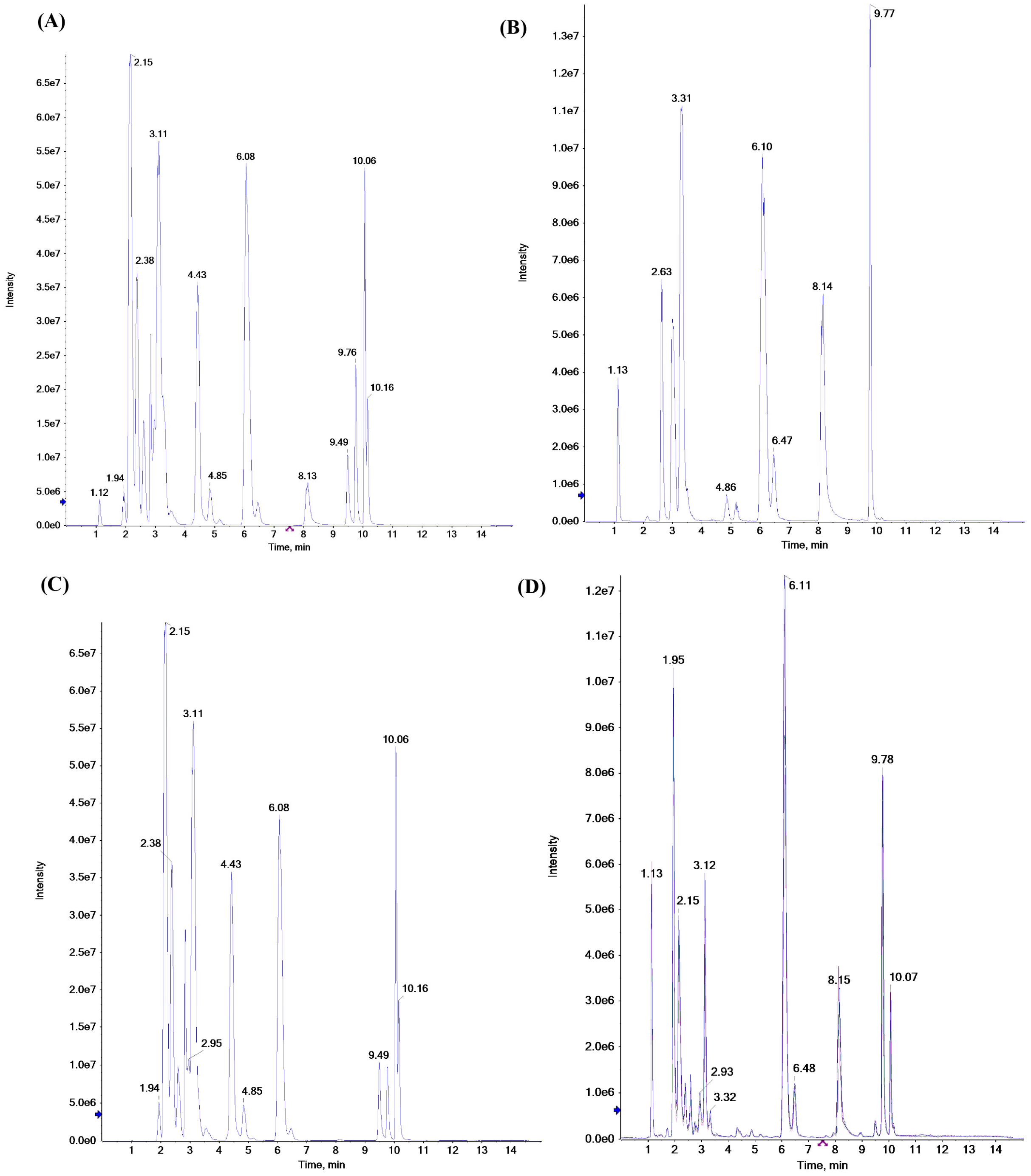

Figure S2. Ions current diagram. (A) Total ion current diagram (standard), (B) Extraction of negative ion current, (C) Extraction of positive ion current diagram, (D) Total ion current diagram (QC sample superposition).
