# Supplementary material for: Comparison of Metabolites and Main Nutritional Components between Uncooked and Cooked Purple Rice
Source: Metabolites. 2023 Sep 15;13(9):1018. doi: 10.3390/metabo13091018 (PMC10536460; doi:10.3390/metabo13091018)
Supplement: Supplementary file 1 [file metabolites-13-01018-s001.zip › Figure S3.pdf]

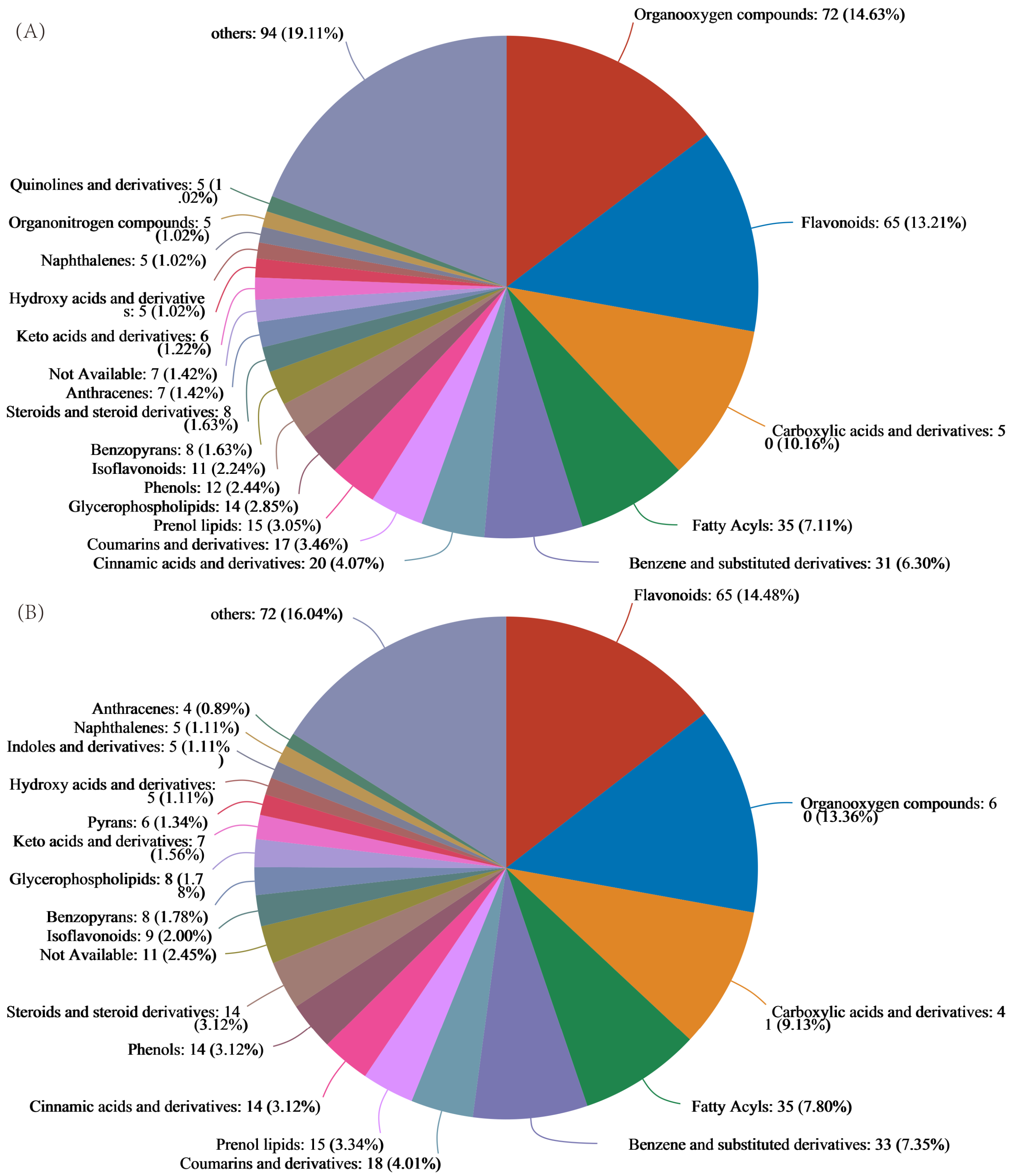

Figure S3. Statistical map of compounds. The name and percentage of the metabolites in the selected HMDB hierarchy (Class) are displayed in a high to low order, depending on the number of metabolites. The different colours in each pie plot in the figure represent a different HMDB classification, the area of which represents the relative proportion of the metabolites in that classification. (A) YZ6C vs YZ6U, (B) YZN1C vs YZN1U.
