# Supplementary material for: Comparison of Metabolites and Main Nutritional Components between Uncooked and Cooked Purple Rice
Source: Metabolites. 2023 Sep 15;13(9):1018. doi: 10.3390/metabo13091018 (PMC10536460; doi:10.3390/metabo13091018)
Supplement: Supplementary file 1 [file metabolites-13-01018-s001.zip › Table S1.pdf]

Table S1. The linear equations for the flavonoids and phenolic compounds.

Metabolites	Retention time	limit of detection	limit of quantitation	Standard curves	RSD (%)
4-Hydroxyproline	4.42	28.65	28.65	y = 1387.24124 x + 19492.53618 (r = 0.99891, r^2 = 0.99781)	5.93
beta-Alanine	3.59	40.65	40.65	y = 154.99409 x + 2159.95738 (r = 0.99895, r^2 = 0.99791)	10.09
gamma-Aminobutyric acid	2.59	47.48	236.23	y = 1284.10741 x + 1.64968e5 (r = 0.99534, r^2 = 0.99071)	7.73
L-Alanine	4.31	59.29	59.29	y = 91.37700 x + 3853.61512 (r = 0.99970, r^2 = 0.99941)	12.32
L-Arginine	9.77	210.57	210.57	y = 671.72717 x + 1.07202e5 (r = 0.99412, r^2 = 0.98828)	4.44
L-Asparagine	6.47	92.69	113.09	y = 65.65557 x + -4026.71185 (r = 0.99915, r^2 = 0.99829)	9.68
L-Aspartic acid	8.14	852.21	1107.14	y = 717.14176 x + -5.95497e5 (r = 0.99884, r^2 = 0.99769)	7.92
L-Glutamic acid	6.1	24.16	142.26	y = 86.70728 x + 1136.28475 (r = 0.99971, r^2 = 0.99942)	6.94
L-Glutamine	6.08	949.57	2293.01	y = 1229.07338 x + -1.12828e6 (r = 0.99714, r^2 = 0.99428)	13.21
L-Glycine	5.18	569.93	569.93	y = 5.01053 x + -1425.62346 (r = 0.99883, r^2 = 0.99766)	7.99
L-Histidine	9.49	32.69	32.69	y = 834.62443 x + -726.29643 (r = 0.99965, r^2 = 0.99929)	10.00
L-Isoleucine	2.43	8.61	942.49	y = 1624.94987 x + 3.51899e4 (r = 0.99675, r^2 = 0.99351)	8.99
L-Leucine	2.21	127.70	277.79	y = 33.37250 x + -2505.92657 (r = 0.99876, r^2 = 0.99752)	10.00
L-Lysine	10.08	1332.43	2308.00	y = 467.30294 x + -6.15385e5 (r = 0.99917, r^2 = 0.99835)	11.12
L-Methionine	2.65	29.40	29.40	y = 384.48336 x + 2477.42204 (r = 0.99927, r^2 = 0.99854)	2.05
L-Ornithine	10.16	36.82	36.82	y = 266.97790 x + -1431.05877 (r = 0.99803, r^2 = 0.99606)	10.83
L-Phenylalanine	2.16	44.21	72.46	y = 2950.84594 x + -5.00651e4 (r = 0.99857, r^2 = 0.99714)	11.23
L-Proline	3.11	26.92	26.92	y = 14549.47721 x + 20496.44559 (r = 0.99887, r^2 = 0.99774)	15.81
L-Serine	6.2	4.31	4.31	y = 137.68201 x + 4149.38611 (r = 0.99842, r^2 = 0.99685)	12.10
L-Threonine	4.85	48.74	73.69	y = 307.07632 x + -5473.33944 (r = 0.99930, r^2 = 0.99859)	3.04
L-Tryptophan	2.13	153.21	539.68	y = 3727.27793 x + -4.72987e5 (r	9.82

				= 0.99728, r^2 = 0.99456)	
L-Tyrosine	2.99	5.17	415.03	y = 409.62858 x + 28203.17163 (r = 0.99817, r^2 = 0.99635)	11.98
L-Valine	2.9	142.71	142.71	y = 2206.36574 x + -5.56010e4 (r = 0.99934, r^2 = 0.99868)	8.22
Taurine	3.31	25.12	25.12	y = 2922.15735 x + 3469.61469 (r = 0.99735, r^2 = 0.99470)	7.29
