# Supplementary figures and images for: Comparison of Metabolites and Main Nutritional Components between Uncooked and Cooked Purple Rice
Source: Metabolites. 2023 Sep 15;13(9):1018. doi: 10.3390/metabo13091018 (PMC10536460; doi:10.3390/metabo13091018)

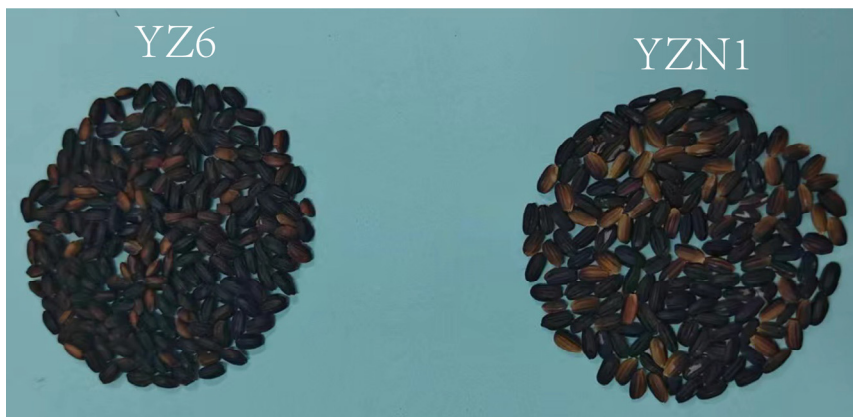

**Figure S1.** Two purple rice pictures.

Supplement: Supplementary file 1 [file metabolites-13-01018-s001.zip › Figure S1.pdf]
